# X-ray Ptychography Imaging of Human Chromosomes After Low-dose Irradiation

**DOI:** 10.1007/s10577-021-09660-7

**Published:** 2021-03-31

**Authors:** Archana Bhartiya, Darren Batey, Silvia Cipiccia, Xiaowen Shi, Christoph Rau, Stanley Botchway, Mohammed Yusuf, Ian K. Robinson

**Affiliations:** 1 London Centre for Nanotechnology, University College, London, UK; 2 Department of Chemistry, University College, London, UK; 3 Research Complex at Harwell, Harwell Campus, Didcot, UK; 4 Diamond Light Source, Harwell Campus, Didcot, UK; 5 Department of Physics, New Mexico State University, Las Cruces, NM 88003 USA; 6 Centre for Regenerative Medicine and Stem Cell Research, Aga Khan University, Karachi, Pakistan; 7 Condensed Matter Physics and Materials Science Division, Brookhaven National Lab, Upton, NY 11973 USA

**Keywords:** X-ray imaging, X-ray microscopy, Karyotype, Irradiation, Chromosome structure, Mass determination

## Abstract

Studies of the structural and functional role of chromosomes in cytogenetics have spanned more than 10 decades. In this work, we take advantage of the coherent X-rays available at the latest synchrotron sources to extract the individual masses of all 46 chromosomes of metaphase human B and T cells using hard X-ray ptychography. We have produced ‘X-ray karyotypes’ of both heavy metal–stained and unstained spreads to determine the gain or loss of genetic material upon low-level X-ray irradiation doses due to radiation damage. The experiments were performed at the I-13 beamline, Diamond Light Source, Didcot, UK, using the phase-sensitive X-ray ptychography method.

## Introduction

Chromatin, composed of DNA-protein complexes present in nucleated cells, is the chemical description of chromosomes. They form a thread-like structure during interphase which becomes condensed at metaphase stage immediately preceding cell division. DNA adopts different levels of packaging to fit within the approximately 10 μm nucleus of the cell. In each chromosome, a ~2-m-long linear strand of DNA is wrapped around an octamer of histone proteins (H2A, H2B, H3 and H4) to form nucleosomes, 11 nm in diameter, referred to as ‘beads on a string’ (Olins and Olins, 1974; Travers, 2014). Histone H1 proteins are linkers that form complex structures by linking nucleosomes together to maintain chromatin stability (Maeshima & Eltsov, 2008). The nucleosomes are the basic building blocks of the chromosome structure.

The diploid (2-copy) human genome consists of slightly more than 6.4 billion base pairs (bp) (Goldfeder et al., 2017) located within 22 pairs of autosome chromosomes and one pair of sex chromosomes (Brown, 2002). The chromosomes are numbered according to their size determined by flow cytometry (Harris et al., 1986). Chromosome 1 is the largest and has a 248,956,422 base pairs (per copy) and the smallest chromosome is made up of 46,709,983 base pairs. In the whole human genome, only 30,000–40,000 genes (information carrying fragments of DNA) are protein-coding regions of DNA, while the remainder have structural or repository functions or are otherwise thought to be inactive (Lander et al., 2001).

Understanding the organisation of chromatin into higher-order structures and the associated condensation process remains one of the key challenges in structural biology. This has particular implications for gene transcription at the molecular level, concerning how the densely packed DNA is efficiently uncoiled for transcription, for example. Chromatin is found to supercoil to form a high-order structure known as the 30-nm chromatin fibre that then folds into a compact mitotic chromosome (Tremethick, 2007) (Maeshima et al., 2014). The compaction ratio for the formation of each nucleosome is 1:6. For a 30-nm chromatin fibre, it is 1:36 and for a full mitotic chromosome it is >1:10,000 (Strachan & Read, 2004). The 30-nm structure has been reported as either Solenoid or Zigzag (Maeshima et al., 2014), based on their unique coiling patterns and is still controversial. Other factors such as monovalent and divalent cations play important roles in regulating the high-order structures of chromosomes. The concentration of monovalent Na^+^/K^+^ and divalent Ca^2+^/Mg^2+^ is found to increase from interphase to metaphase in the cell cycle (Maeshima & Eltsov, 2008).

A karyotype is a way of classifying the full genetic complement, in which chromosomes are arranged according to their size and shape. This is the first stage of identifying any genetic anomaly and aberrations, such as structural and numerical aberrations, aberrations, generally in any organism. Experimentally, chromosomes at the metaphase or prometaphase stage of the cell cycle are arrested and stained with an appropriate dye for karyotyping (Ried et al., 1998) (Anderson et al., 2002). Karyotyping has efficient clinical applications and can be used for diagnosing genetic diseases (Cram et al., 1988) (Grimwade et al., 2010).

We can estimate the protein composition of the human metaphase chromosomes, knowing they are composed of DNA-protein complex. From the human genome literature, the amount of DNA present in each individual human chromosome (Lander et al., 2001) (Piovesan et al., 2019) is known but the protein composition of metaphase chromosome is still less clear. We note furthermore that the human genome consists mainly of the coding sequences and that the sequencing methods are unable to resolve the repeated sequences. Consequently, the DNA masses based on the human genome sequence will be underestimated. This is most likely the case for chromosomes 1, 9, 15 and 16 that are heteromorphic and contain heterochromatin-rich regions.

In this paper, we investigate the amount of protein (histone and non-histone) present in each individual metaphase chromosome from the obtained masses using the new imaging method of X-ray ptychography. This measures the phase shifts of X-rays passing through the sample in a very quantitative way by merging coherent diffraction patterns from overlapping regions of the sample self-consistently. Because the phase shift can be calibrated in units of electron density, the total number of electrons in each isolated chromosome, and hence its mass, can be determined. When the image contains a full ‘spread’ of 46 human chromosomes in metaphase, their individual identities can be established in the form of a mass karyotype. We then use the new imaging method to investigate the effects of prior low-dose irradiation applied to living cells.

We know the number and the composition of the histones from the Protein Data Bank (PDB) that are known to be associated with every 166 base pairs (bp) of DNA, wrapped around the protein core to form each nucleosome, which forms the fundamental structural subunit of chromatin fibre, the DNA-protein complex. A single nucleosome contains eight histones, two each of H2A (14,135 Da), H2B (13,906 Da), H3 (15,404 Da) and H4 (11,367 Da), attached to 146 bp of DNA and one linker histone H1 (~32,000 Da), occupying ~20 bp, which connects two nucleosomes to form a chain of chromatin (Harshman et al., 2013). This gives 141,624 Da of histone protein per nucleosome. Secondly, we calculated the mass of the 166 bp of DNA, using the average molecular weight (660 Da) of a base pair (Bench et al., 1996). Using 1 Da = 1.66×10^−27^ kg, this gives the true physical mass of the nucleosome (Doležel et al., 2003).

We need to include the essential, additional non-histone proteins (condensins, topoisomerases, etc.) in the calculation too. Uchiyama et al. (2005) measured the breakdown of all proteins present in meta-phase chromosomes. They found that histone proteins represent 60% of the total, so the non-histone component can be estimated to be the fraction 40/60 (non-histones/histones) or 94,416 Da per nucleosome. Consequently, the estimated mass of one complete nucleosome can be calculated from its three important components, DNA with 109,600 Da, histone protein with 141,624 Da and non-histone protein with 94,416 Da with a total of 345,640 Da per nucleosome.

If the total mass of a single nucleosome is 3.5×10^5^ Da, the mass of chromosome 1, with its 248,956,422 base pairs, can be estimated by first calculating the number of nucleosomes as 2.5×10^8^/166 = 1.50×10^6^, giving a physical mass of 1.50×10^6^ × 3.5×10^5^ × 1.66×10^−27^ kg = 0.87 pg. The DNA alone would be 0.28 pg with the remaining 0.59 pg being the protein. These calculations relate to the dry mass and do not include water of solvation or any bound ions. From the mass of the nucleosome, the expected masses for all 46 human chromosomes can be calculated from the DNA content documented by the Human Genome Project (Lander et al., 2001). Therefore, in this manner, we can calculate the expected mass of all 46 chromosomes for both the cell types investigated in this work.

## X-ray imaging and ptychography

The strength of X-ray penetration directly leads to the choice of X-ray wavelength used for high-resolution imaging of biological samples (Larabell & Nugent, 2010). Detailed information can be obtained from atomic to molecular level without any sample processing such as fixing, staining or sectioning (Robinson et al., 2016). X-ray microscopy is sensitive to matter because a photon from an X-ray source interacts with the inner shell of each atom of the specimen through direct absorption or refraction and change of phase (Howells et al., 2006). The complex refractive index, *n* =1 − **δ** + iβ, describes the X-ray interaction with matter, with the real component (**δ**) describing the phase change and the imaginary component (β) denoting the absorption of the beam (Hémonnot & Köster, 2017).

X-rays used for imaging biological samples are usually divided into soft X-ray and hard X-ray depending upon their wavelength and photon energy (Chapman & Nugent, 2010). The wavelength range for soft X-rays is 2–5 nm to operate in the ‘water window’ of the spectrum between the carbon K-edge at 284 eV and the oxygen K-edge at 540 eV (Maser et al., 2000). Soft X-rays are claimed to be advantageous over hard because they protect against sample mass loss and morphological distortion even at a radiation dose of 10^10^ Gray (1 Gy=1 J/Kg) and can image frozen hydrated biological samples maintaining a structure close to the native state (Maser et al., 2000). The technique was used for investigating ultrastructure of a cryopreserved female inactive X-chromosome by soft X-ray tomography (SXT) in combination with cryogenic fluorescence microscopy by Smith et al. (2014). In addition, a frozen hydrated yeast cell was resolved at a resolution of ~25 nm using soft X-ray coherent diffraction imaging by Huang et al. (2009).

Scanning X-ray fluorescence microscopy has been used to map the signal of iron (Fe), phosphorus (P) and sulphur (S) in human nuclei, in which P and S are presumed to be associated with the DNA and protein components of the nuclei (Robinson et al., 2016). The first ever attempt to image micron-sized details of high-order structure of chromosome by Robinson et al., (2015) made use of the SACLA X-ray Free Electron Laser, where 400,000 diffraction patterns were collected.

Using hard X-rays with their high penetrating capacity, a spatial resolution of ~1 μm was achieved by Yamamoto and Shinohara (2002). This gives better contrast images and does not require invasive staining. Third-generation synchrotron radiation sources are used to generate photon energies between 5 and 10 keV to image biological samples with a thickness exceeding the 100-nm limit of transmission electron microscopy (TEM) (Guk et al., 2008). More importantly, ‘lensless’ methods exploit the high coherence of third-generation synchrotron radiation sources to solve the ‘phase problem’ to provide an electron density map by phase contrast (Nishino et al., 2009). Soft X-rays tend to give absorption information instead (Shapiro et al., 2005). While soft X-rays are limited in resolution, they are claimed to prevent radiation damage (Kirz et al., 1995). Hard X-rays are necessary to penetrate thick samples, like chromosomes, that are too thick for TEM (Shemilt et al., 2015).

A major breakthrough was the successful X-ray Coherent Diffraction Imaging (CDI) study of an unstained human metaphase chromosome by Nishino et al. (2009) at a photon energy of 8 keV at the Spring8 synchrotron facility in Hyogo, Japan. The obtained diffraction patterns were processed using a hybrid input output (HIO) phase retrieval algorithms by Nishino et al. (2009). The axial structure was resolved in 2D and 3D with a resolution of 144 nm but not the internal structure of the chromosome (Nishino et al., 2009).

Going beyond CDI, ptychography is a coherence-based technique in which an object is illuminated and scanned in a step-wise fashion to produce an array of diffraction patterns from partially overlapped probe spots on the object. The obtained diffraction patterns are run through an iterative reconstruction algorithm, expanded from those used for single-shot CDI data (Rodenburg et al., 2007) (Maiden & Rodenburg, 2009). As with CDI, the ptychography method takes advantage of the ‘Nyquist-Shannon Theorem’ of oversampling (Chapman et al., 2006, Miao et al., 1998) in each diffraction pattern. Overlapping illumination spots provides additional information density and ptychography tends to be more forgiving of experimental restrictions such as data noise than single-shot CDI.

The output of ptychography is a pair of images: the amplitude measures the extent of X-ray absorption by the sample and the phase measures the phase delay introduced to the beam due to refraction as it passes through the sample. The combination of high penetration power of hard X-rays and sensitivity of CDI is the main benefit of hard X-ray ptychographic imaging. It has the capability for nanometre-scale resolution of three-dimensional structures with a high sensitivity to electron density changes (Dierolf et al., 2010). A reconstruction algorithm, called the Ptychographical Iterative Engine (PIE), is used to solve the phase problem; however, it has the drawback of requiring prior knowledge of the illumination function or wave front, called the ‘probe’ (Rodenburg et al., 2007). The improved ‘extended PIE’ (ePIE) algorithm is more general in determining both the object and the complex probe functions (Maiden et al., 2017). Both PIE and ePIE algorithms iterate between real and reciprocal space applying constraints in both spaces until a solution is reached.

Previous work on biological samples using ptychography includes freeze-dried diatoms at a resolution of 30 nm, bacteria at a 20-nm resolution, frozen-hydrated yeast at a 85 nm resolution and 3D nonporous glass at a resolution of 30 nm (Deng et al., 2015). Corelative imaging of X-ray fluorescence microscopy (XFM) and X-ray ptychography were used to image whole frozen-hydrated *C. reinhardtii* algal cells to map the elemental constituent in a 3D fashion by including tomography to obtain a 3D view of the cells (Deng et al., 2019). XFM was used to get fluorescence signal from available element and ptychography for spatial resolution and the acquired resolution was ~2 μm. Soft X-ray ptychography has been applied to obtain chemical information from ~ 2-μm-thick freeze-dried *Deinococcus radiodurans*, without slicing and staining at a photon energy of 512 eV in the water window (Beckers et al., 2011). X-ray ptychography has been demonstrated to obtained quantitative information of human metaphase chromosome to build a preliminary karyotype on the basis of mass (Shemilt et al., 2015), and recently to image a human nucleus under cryogenic condition (Yusuf et al., 2017).

## Radiation effects on chromosomes

Ionising radiation (IR) causes a wide spectrum of lesions including DNA single-strand breaks, double-strand breaks (DSBs), base damage, and DNA-protein cross-links (Balajee et al., 2014; Nakano et al., 2017). Among these, DSBs are considered to be lethal and most likely to lead to cell killing (Botchway et al., 1997). This is most probably because the non-repairable DSBs or mis-repaired fractions lead to chromosomal aberrations (CAs) (Balajee et al., 2014). Aberrations can also be caused by external factors like ultraviolet (UV), exposure to sunlight, ionising radiation (X-rays, gamma rays) and toxic chemicals, generally depending on radiation quality (Durante & Formenti, 2018), dose (Touil et al., 2000) and dose rate (Fujimichi & Hamada, 2014; Lowe et al., 2020). Additional factors like unequal cell divisions, replication errors and the enzymatic reactions can also cause aberrations (Jain et al., 2017). Exposure to radiation can cause simple or clustered DNA damage (Gulston et al., 2004; Magnander et al., 2010). However, one or two aberrations per DNA helical turn leads to double DNA strand breaks (Lomax et al., 2013).

When transversing biomolecules, the X-ray irradiation deposits part of the energy, characterised by tracks. ‘Linear energy transfer’ (LET) deposition results in chemical modifications in DNA (Lomax et al., 2013) (Desouky et al., 2015). Energy absorbed by water causes water radiolysis, producing free radicals of H^+^ and OH^−^ (Azzam et al., 2014), which undergo reactive recombination into species such as toxic superoxide that causes DNA damage (AbdulSalam et al., 2017).

There are two major types of chromosomal aberrations, known as numerical and structural aberrations. Numerical aberrations consist of aneuploidy, due to improper distribution of chromosomes at the anaphase stage of the cell cycle (for example trisomy and monosomy) and triploidy (presence of an extra haploid chromosome) (Tobias, 2011). These defects, known as non-dysjunction, take place at mitosis or later at meiosis phase of the cell division (Tobias, 2011). The structural aberrations occur either due to breakage or irregular reunion, due to exposure to mutagens and/or ionising radiations. These are the kind of aberrations we sought to identify in this study.

## Materials and methods

### Cell cultured B and T cells

B lymphocyte cells from the Yoruba male cell line (GM18507, International HapMap Project) were used for the chromosome studies (Yusuf, Chen, et al., 2014b; Yusuf, Parmar, et al., 2014a) (Shemilt et al., 2015) (Estandarte et al., 2016). The B cells are suspension cells and were used at passage 15 in this study.

Primary T lymphocyte cells were donated by a 22-year-old female, provided by Public Health England (PHE), Oxford, UK. Additionally, lethally irradiated lymphoblastoid cells (GM1899A, provided from PHE) were used as feeder cells. The primary T cells were cultured in the laboratory of PHE. T cells at passage 3 were used in this study. The stimulated human T lymphocytes were prepared from 10 ml of blood, collected into BD Vacutainer® lithium heparin tubes (Becton Dickinson). Five milliliters of Histopaque-1077 (Sigma-Aldrich) pre-warmed to room temperature was aliquoted into four 15-ml conical bottom centrifuge tubes. Ten milliliters of blood was mixed with 10 ml of Hank’s Balanced Salt Solution (HBSS, Life Technologies) pre-warmed to room temperature in a 50-ml conical bottom tube. Five milliliters of diluted blood was layered slowly onto each of the four tubes containing Histopaque-1077 using a sterile ‘pastette’ (Alpha Laboratories, Eastleigh, UK). Tubes were centrifuged at room temperature at 1600 rpm for 20 min. Following phase separation, the top serum layer was aspirated from each tube leaving around 0.5 cm of liquid above the buffy coat cell layer. The buffy coats from sample tubes were collected and transferred into fresh 15-ml tube containing 10 ml of HBSS and mixed by inverting 5 to 6 times. The tubes were centrifuged at room temperature at 1200 rpm for 5 min. The supernatant was aspirated followed by re-suspending cell pellet in 5 ml of HBSS. Suspended cells were combined into one tube and centrifuged again at room temperature at 1200 rpm for 5 min. After the supernatant was aspirated, the cells were washed twice with 10 ml of HBSS and a 20-μl aliquot of cell suspension was taken for cell counting. The tube was centrifuged at room temperature at 1200 rpm for 5 min and the supernatant was aspirated. Next the cells were re-suspended at a concentration of 3×10^6^ cells/ml in freeze mix, then transferred to cryogenic vials in Mr. FrostyTM container and frozen at −80 °C.

A cryovial containing 3×10^6^ cells/ml, passage 3, was thawed at 37 °C in a water bath for 2 min. The cells were transferred to a 15-ml conical bottom tube containing 10 ml of stimulating growth medium (SR10) containing RPMI 1640 (Dutch modification) supplemented with 10% heat inactivated FBS, 1 mM sodium pyruvate, 2 mM l-glutamine, 100 U/ml penicillin, 100 μg/ml streptomycin, 50 μM 2-mercaptoethanol (GIBCO/Life Technologies), 20 U/ml recombinant interleukin-2 (IL2; Sigma-Aldrich) and 0.4 μg/ml phytohaemagglutinin (PHA; Sigma-Aldrich). Cells were mixed by inverting the tube and centrifuged at room temperature for 5 min at 1200 rpm. The supernatant was aspirated, cell pellet was re-suspended in 10 ml of SR10 and centrifuged at room temperature for 5 min at 1200 rpm.

Prior to mixing feeder cells into T lymphocytes, a cryovial of feeder cells was thawed in the water bath at 37 °C for 2 min. The supernatant from T lymphocytes was aspirated and the cells were re-suspended in 10 ml of SR10. The feeder cells were transferred into a 15-ml conical bottom tube containing T lymphocytes, mixed by inverting the tube several times and centrifuged at room temperature for 5 min at 1200 rpm. The supernatant was aspirated and the cell pellet was re-suspended in 10 ml of SR10. The cell suspension was transferred to a vented 25-ml flask and incubated at 37 °C with 5% CO_2_ at an angle of about 10° from the horizontal. Cells were left undisturbed for 4 days and thereafter they were disaggregated and counted daily using an ADAM cell counter (Labtech International Ltd., Uckfield, UK). When the cells reached a density of 0.8×10^5^ cells per ml, they were diluted 1:2 with growth medium (GR10) that contained SR10 media without PHA.

### Irradiation of T cells

Confluent T lymphocytes cells, 5×10^6^ cells/ml in a T75 vented cell culture flask, were transported to PHE for X-ray irradiation. Cells were irradiated with X-rays from the A.G.O. X-ray System, model CP160/1 (AGO X-RAY Ltd., Martock, UK) located at PHE using an X-ray energy of 250 kVp (maximum voltage applied across an X-ray tube during the creation of X-rays within the X-ray system) with a half value layer of 2 mm of copper and aluminium compound filters and 13-mA current, giving a dose rate of 0.5 Gy/min. Three out of four flasks with 11 ml cell media, keeping one flask as a control, were irradiated with a different radiation doses such as 0.1 Gy, 0.5 Gy and 1 Gy with a duration of 12 s, 1 min and 2 mins respectively. After irradiation, 9 ml of fresh SR10 media was added in each flask and mixed well by shaking manually. Thereafter, to arrest chromosomes at their mitotic (most condensed) stage, colcemid (Karyomax, Gibco by Life technologies (10 μg/ml)) was added at the final concentration of 0.2 μg/ml into each flask. The flask was kept for 16 h in the incubator at 37 °C with 5% of CO_2_ before harvesting.

### Preparation of spreads

Metaphase chromosomes were prepared according to previously published protocols (Moralli et al., 2011; Yusuf et al., 2014a, b) from both B and T lymphocytes. To arrest chromosomes at their mitotic stage at a different cell cycle, colcemid (10 μg/ml) was added for 16 h at the final concentration of 0.2 μg/ml in each flask before harvesting. The supernatant from each centrifuged tube was aspirated followed by addition of 6 ml pre-warmed (37 °C), hypotonic solution (KCl, 75 mM) which was slowly added in the falcon tubes. The tubes were immediately transferred to the pre-warmed water bath at 37 °C for 8–10 min and then spun at 1000 rpm for 10 min. Meanwhile, a prepared fresh methanol:acetic acid solution (MAA) was prepared in the ratio of 3:1 to fix the extracted chromosomes. After the supernatant was aspirated, 6 ml of MAA was quickly added dropwise and shaken immediately to dislodge the pellet in each tube. The tubes were spun at 1000 rpm for 10 min and then the supernatant was aspirated. The washing procedure with MAA was repeated three times to get clear solutions of chromosomes from both T and B lymphocytes. The prepared chromosome solutions were stored at −20 °C for future use.

Windows with chromosomes were prepared according to previously published protocols (Robinson et al., 2015; Yan et al., 2016). Two different-sized silicon nitride membrane X-ray windows (Silson Ltd.) were used. These had 0.25×0.25 mm^2^ window opening, membrane thickness 30 nm and 100 nm or 0.50×0.50 mm^2^ window opening, membrane thickness 30 nm and 100 nm. In each case, the silicon nitride membrane was located at the centre of a 3-mm octagonal silicon frame of thickness 200 μm. With the flat side of the membrane facing upward, the membrane windows were mounted on a parafilm-wrapped glass slide and placed inside the air chamber of an automated GloQube glow discharge system (Quorum Technologies Ltd., Sussex, England) designed for negative hydrophilisation of TEM grids. The treatment was carried out for 30 s at 30 mA current. This treatment causes chromosomes in suspension to adhere evenly on the surface of the silicon nitride windows.

Once the grids were made hydrophilic, 5 μl of MAA fixed chromosomes was dropped immediately from a height of 10 cm to get well-scattered metaphase chromosome spreads. These were left to air-dry, then validated using an Olympus LEXT laser scanning confocal microscope in combination with custom analysis software, LEXT. A ×10 objective was used for localisation of spreads and ×20 or ×50 were used for mapping of the locations of well-formed chromosome spreads. These optical images were saved for reference alignment and subsequent localisation of the spreads during the X-ray imaging.

One-hundred-fifty-nanometer gold nanoparticles (Sigma-Aldrich) were used as a test pattern (fiducial markers) for X-ray imaging. Two microlitres of gold nanoparticles was dissolved in 8 μl of MilliQ water and then dropped (3 μl) on the silicon window (0.50 mm × 0.50 mm, membrane thickness 30 nm) and left to dry before imaging at room temperature.

### Multiplex fluorescence in situ hybridization

For identification of any chromosomal aberrations, the conventional karyotype was first performed on non-irradiated chromosomes obtained from both the T and B lymphocytes with the help of chromosome identification technique known as multiplex fluorescence in situ hybridization (M-FISH). The chromosomes were stained with a 24XCyte probe kit which labels all 24 chromosomes with a 5 different fluorochromes arranged in a combinatorial manner. This helps to visualise the structural rearrangements (e.g. translocations) of the chromosomes and any numerical aberrations (Anderson et al., 2002) (Balajee et al., 2014).

**Fig. 1:**
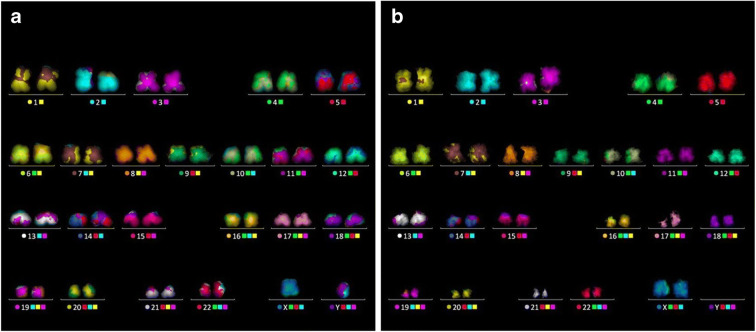
M-FISH karyotype performed on non-irradiated chromosomes obtained from **a** B lymphocytes, a male cell line (1-22, XY chromosomes), and **b** primary T lymphocytes from a female donor (1-22, XX chromosomes)

The M-FISH karyotype in Fig. 1 shows a set of 22 pairs of autosome chromosomes and one pair of sex chromosomes in each, depending upon the gender. The chromosome numbers are allocated their colours, coded by the computerised-colour-code scheme of MetaSystems, and placed above the number assigned to each colour (Anderson, 2010; Speicher et al., 1996). The karyotype helps to identify the stability and the quality of the chromosomes obtained from B and T lymphocytes. After validation, the same preparation of chromosomes was used for the X-ray ptychography imaging, using the optical image as an index to locate the well-formed spreads.

### Heavy metal stains

Two types of heavy metal stains were used for staining the chromosomes, once placed on the silicon nitride windows:

- Platinum blue, synthesised in our laboratory (Yusuf, Chen, et al., 2014b; Yusuf, Millas, et al., 2014c) at Research Complex at Harwell (RCaH), Oxford, UK, at 6 mM concentration: 0.015 g of platinum blue powder was dissolved in 5 ml of MilliQ water and stored at 4 °C.
- Uranyl acetate (UA) which is a negative stain, was prepared at 1% from a stock solution of 2% UA (Taab Laboratories Equipment Ltd.), in equal proportion of milliQ water and stored at 4 °C.

Chromosome samples were mounted on the silicon nitride windows and, once dry, they were stained either with 6 mM platinum blue or 1% uranyl acetate. To stain with platinum blue, 2 to 3 μl of 6 mM platinum blue solution was dropped on the silicon windows containing chromosome spreads at room temperature. Excess dye was blotted off using Whatman filter paper and left to air-dry. The dried grids were stored in a grid box.

To stain with 1% UA solution, the grids containing chromosome spreads were first washed twice, for 30 s each wash, in MilliQ water and then stained twice, for 30 s each with a drop of 1% UA solution. After every wash and staining, the grids were blotted using Whatman filter paper. After the last stain cycle, the sample was blotted for longer time, 1 min, to get rid of excess UA. This staining was performed in a radioactive laboratory following full safety and training procedures. The stained chromosome samples were stored in the radioactive laboratory for 24 to 48 h before taking them to the beamline for X-ray imaging.

The chromosomes mounted on silicon nitride windows were loaded into the sample holders. We used either a single grid holder prepared on SEM stubs in our laboratory or a 4 × 4 array 3D printed holder from I-13 beamline, depending on the number of samples to be imaged at a time. In the 4 × 4 array holder, the recessed holes fit the 3-mm grids, which are captured by the protruding ring of the mating part. The lid of the holder was screwed on carefully to avoid cracking the grids.

### Experimental setup for X-ray ptychography

**Fig. 2:**
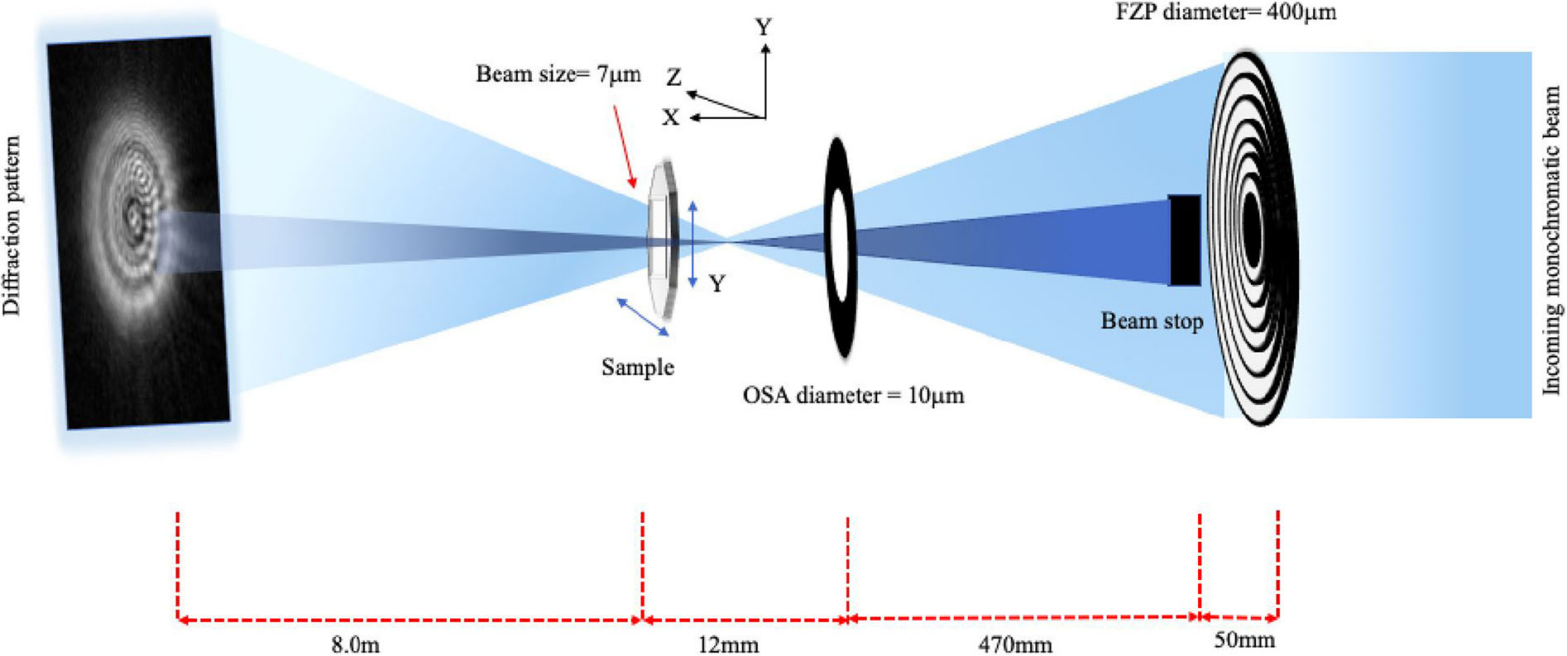
Schematic diagram of the beamline setup at I-13-1 Diamond light source for X-ray ptychography imaging

Beamline I-13 has a multimodal end-station for ptychographic imaging. For this study, the X-ray beam was filtered through the double-crystal Si(111) monochromator producing a 9.7 keV photon beam with ∆E/E bandwidth of 10^−4^. The collimated and energy filtered beam was focused using a Fresnel zone plate (FZP) of diameter 400 μm and outer zone width of 150 nm. A 50-μm order sorting aperture (OSA) further downstream was centred to allow only the first diffraction order from the FZP to pass through. A gold central stop (CS), 60 μm in diameter, was used to eliminate the transmitted zero-order beam from passing through the OSA. The sample was positioned out of the focal plane of the FZP, such that there was a 6-μm spot size at the sample plane, selected to match the detector sampling condition required. The sample was mounted on a high-precision piezo stage and scanned perpendicular to the optical axis in a snake-wise stepping raster fashion. A helium gas pipe was placed between the sample and detector to minimise the air scattering so that sample scattering signal could be maximised. The X-ray photon-counting Excalibur detector (Marchal et al., 2013) consisting of 48 Medipix3 chips, in a 1806×1548 pixel array with pixel size 55×55 μm, recorded the X-ray diffraction pattern 8 m downstream from the sample. A schematic of the experimental setup is illustrated in Fig. 2. The raw data are inverted into images through a ptychographic phase retrieval step, performed using the ePIE operator in the PtyREX software package (Batey, 2014) (Maiden & Rodenburg, 2009) (Rodenburg et al., 2007).

**Fig. 3:**
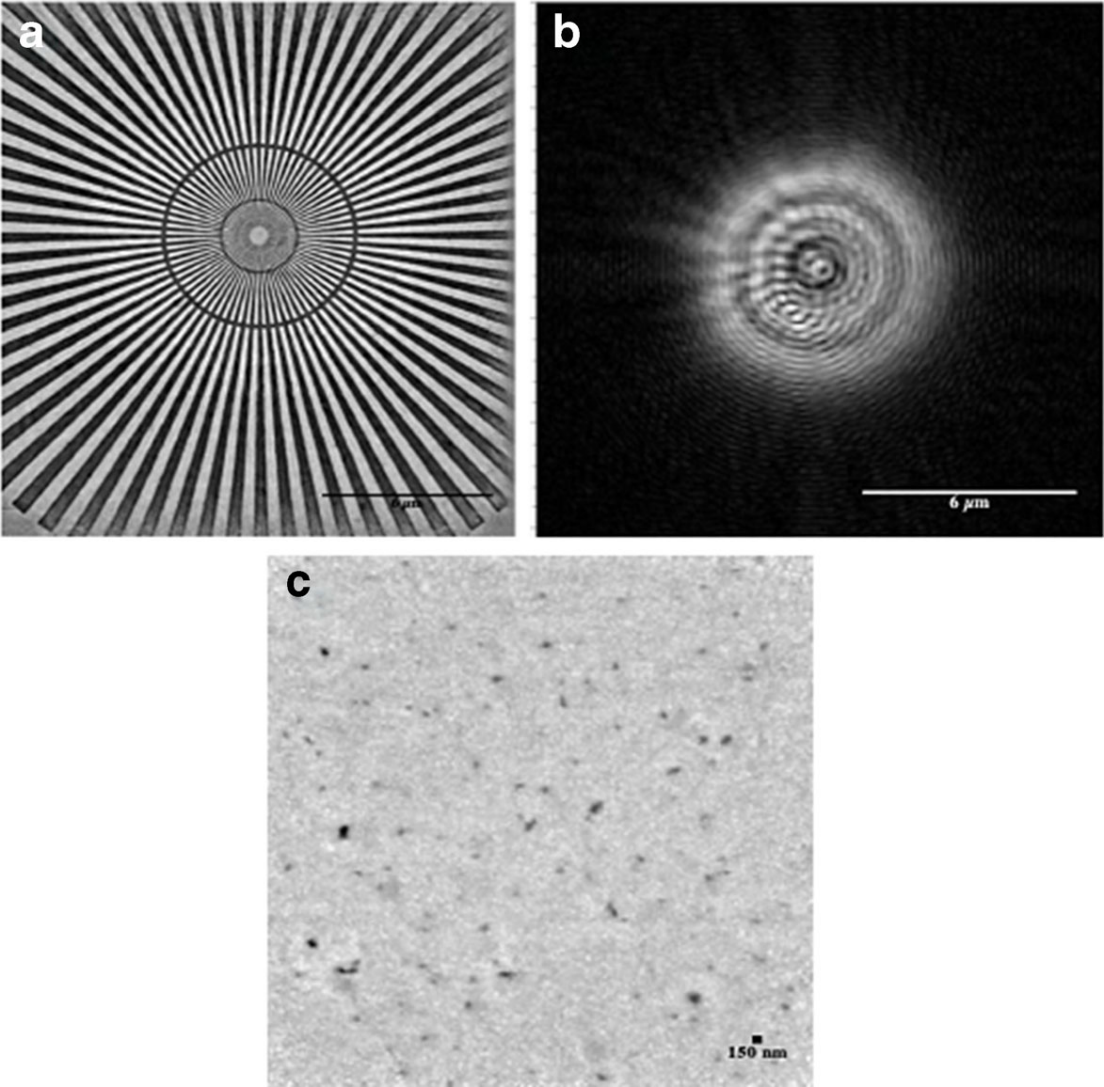
Test sample images used for calibration of the X-ray ptychography **a** reconstructed image of a ‘Siemens star’ standard for measurement of spatial resolution. Field of view (FoV): 64×64 μm^2^, X-ray exposure time: 0.01 s, scale bar = 6 μm. **b** Amplitude of the X-ray beam illumination function (‘probe’) which is the modulus of the complex wave function, scale bar = 6 μm. **c** Reconstructed phase image of 150-nm gold nanoparticles for characterisation of the effectiveness of the phase contrast capability of ptychography. FoV 32×32 μm^2^ and exposure time was 0.1 s per frame

In order to first characterise and optimise the X-ray ptychographic setup, we imaged a ‘Siemens star’ test target. The standard has spokes constructed from 500-nm-thick gold on a SiN membrane; the spacings between the spokes grow radially from 50 nm at the centre to 2 μm at the outer edge. The strong contrast in the high-quality reconstruction of the test sample provided an accurate determination of the illumination function (the ‘probe’), which is the amplitude and phase of the X-ray beam scanned across the sample as shown in Fig. 3. This known ‘probe’ was used later to seed for the reconstruction of the much weaker scattering chromosome samples. As a further calibration, 150-nm gold nanoparticles were also scanned and reconstructed in Fig. 3 c.

Due to the very low absorption coefficient of chromosomes at 9.7 keV energy, only the phase component of the reconstruction was used for further analysis. Ptychographic reconstruction images are quantitative; therefore, the phase values extracted from the images can be converted into the absolute number of the electrons in the sample, giving an absolute measure of its mass, as described below. The phase component of the reconstruction was analysed using ImageJ software.

### Statistical analysis and error estimations

Each spread took about 3 h to measure. This was mostly spent cross correlating the optical images to locate well-isolated spreads, then performing a series of quick scans to centre them in the available field of view (FoV) of the ptychographic scan before the final measurement. Since we depended on capturing a full set of 46 chromosomes in order to safely rank the masses into a karyotype, it was important that one or more chromosomes had not strayed far from the FoV that it might be accidentally associated with another spread. We also found that the segmentation step was occasionally ambiguous, making it hard to divide two overlapping or touching chromosomes. For these reasons, more than half of the results had to be discarded and we were sometimes left with just one good example of a well-measured ‘spread’ for a given sample preparation.

Because the beam time at Diamond Light Source was difficult to get and limited in duration to two experimental runs, there was not enough time to measure a large number of ‘spreads’ from each chromosome preparation and do a full statistical analysis of the variations of the masses. We focussed instead on looking at a single series of samples from the irradiation experiments. A better statistical analysis is planned in the future.

Fortunately, we were able to measure the reproducibility of all the major steps of the mass determination itself. The ptychography measurement was repeated three times with different exposures. The images were reconstructed independently and integrated separately to give independent mass karyotypes. When the segmentation was performed independently on each image, only minor changes were found in the assignment of chromosome numbers, mostly in the closely spaced 9-12 region. It was found that, within error, there was no significant mass loss due to X-ray exposure from one measurement to the next. The mass distributions were reproducible with an asymmetric tail on the high-mass side: the mean was higher than the median in all cases except the highest irradiation level, where it reversed. In that highest dose example, where the mass distribution had become changed to a more symmetric one, the observation was reproducible over the three exposure times.

## Results

### Chromosome images by X-ray ptychography

**Fig. 4:**
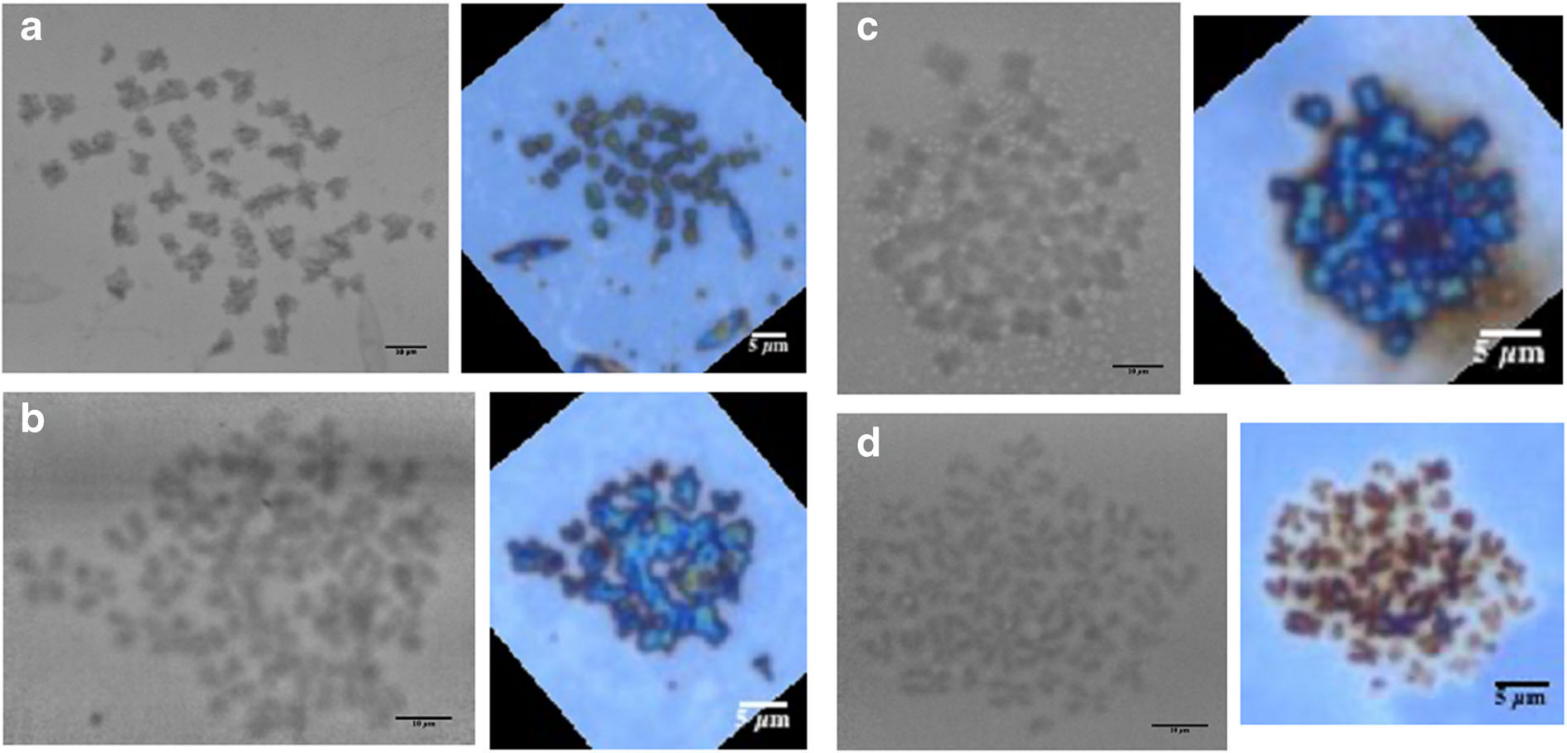
Ptychographic reconstruction of unstained chromosome spreads obtained from T lymphocytes (female cell line). Each panel shows pairs of images, a ptychography phase image (left) and an optical microscope image (right) of the same chromosome spread. Dark regions in the X-ray images represent increase phase shift, with scale bar = 10 μm. Chromosomes appear as blue clustered features in the optical images with scale bar = 5 μm. **a** Unirradiated cells, **b** induced T lymphocytes cells at 0.1 Gy, **c** at 0.5 Gy and **d** at 1.0 Gy. Field of view (FoV) 32×32 μm^2^, with 0.3-s exposure time

Two experimental runs at the I-13 beamline of Diamond Light Source were performed, the first on Yoruba B cells and the second on T lymphocyte cells. The first experiment tested the effects of staining, while the second experiment looked at the samples from irradiated cells to explore the induced damage. The corners of the nitride windows were aligned with the optical images to identify regions containing good-looking spreads. These were mostly scanned with the condensed beam (Fig. 3b) across a scan of 32×32 points with 1-μm steps and an X-ray exposure time of 0.3 s. These data collection schemes allow adequate overlap of the probe positions to solve the phase problem (Batey, 2014). The samples were imaged at room temperature, although attempts to handle cryopreserved samples have been reported elsewhere (Yusuf et al., 2017). The obtained diffraction patterns were reconstructed using 100 iterations of the ptychographic algorithms. Results are shown in Fig. 4 for unirradiated cells and three X-ray radiation doses, 0.1 Gy, 0.5 Gy and 1 Gy, from the second experimental run at I-13. These doses were used to induce live cultured cells, prior to extracting the chromosomes before imaging, to understand the structural rearrangement and change in the individual masses of the chromosomes.

The X-ray phase shifts recorded in the images of Fig. 4 range up to 0.06 radians, which is a direct measure of the electron density of the chromosomal material at that location. The values recorded in the first experiment were only 0.01 radians. It is noteworthy that the average phase values (and hence mass numbers discussed below) emerging from the second experiment were an order of magnitude higher than those from the first experiment. This discrepancy, which was noticed immediately in the apparent contrast of the raw ptychography images, is not fully explained. The experiments were separate runs at Diamond, about a year apart, and both the measurement and reconstruction method improved considerably over that time. But this would be expected to improve only the efficiency and not the phase contrast values, which should be highly quantitative. The most likely explanation was the significant delay of 5–6 months between the sample preparation, with the samples stored in 3:1 methanol acetic acid in the refrigerator, and the measurement for the first experimental run, during which time some of the chromosome mass may have been ‘lost’ during storage. The quantitative numbers for the second batch, where the samples were freshly prepared, are therefore probably more reliable.

### Determination of masses from the chromosome reconstructions

From the ptychography experimental results, the mass of the individual chromosomes was calculated from the phase value of the reconstructed chromosomes scanned at 9.7 keV. The reconstructed phase image of the chromosome spread was processed in ImageJ using a Gaussian filter and an optimum threshold. The number of electrons, *M*, within an individual chromosome, is obtained by summing up the phase shift values inside each image pixel, counted by *j*, so can be calculated by using the master equation (Als-Nielsen & McMorrow, 2011) (Shemilt et al., 2015):

1 $$ M=\sum \limits_j\frac{\phi_j}{{\uplambda \mathrm{r}}_0} pxpy $$

The summation of the phase shift pixel values ($\sum \limits_j{\varnothing}_j$) across the chromosome area (Shemilt et al., 2015) was calculated using the free hand tool in ImageJ. The open areas of the sample were taken as a background phase value and subtracted from each pixel. The wavelength of X-ray beam was λ = 0.13 nm; the real space pixel size of the 2-dimensional (2D) chromosome images was *p*_*x*_ = *p*_*y*_ = 32 nm or 35 nm (depending upon the experiment) and the classical radius of the electron is *r*_0_ = 2.82 × 10^−6^ nm.

In this way, we calculated the masses of each human chromosome found in the spreads by segmenting the images. Since only light atoms are present in DNA, which all contain the same number of protons, neutrons and electrons, the number of electrons measured by ptychography is precisely half the number of Daltons of mass. Thus, the masses of chromosomes were obtained from the summation of the experimental X-ray phase shift across each pixel of the chromosome images (Als-Nielsen & McMorrow, 2011).

**Fig. 5:**
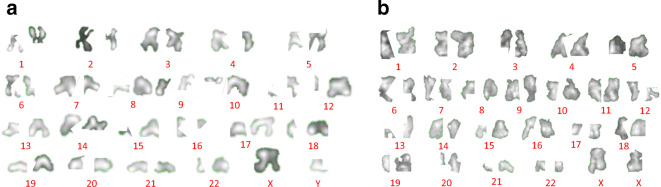
Segmented chromosomes from a single spread, imaged by X-ray ptychography and arranged in order of their individual masses. The chromosome numbers of each homologous pair of chromosomes are indicated following convention (see text). **a** B cells from the Yoruba cell line measured during the first experiment. **b** T cells from the second experiment

The knowledge of the individual masses allows a fully quantitative X-ray karyotype to be generated by ranking them in their chromosome number order. As a first illustrative step, karyotype layout images were prepared in Fig. 5 for direct comparison with the M-FISH results shown in Fig. 1. The chromosomes were segmented using ImageJ following: (i) conversion to a 16-bit images, (ii) subtraction of the background, (iii) adjusting the threshold, (iv) inverted the pixel values by using the ‘Invert Look up Table’ tool and (v) rotating them into place following the convention of the p- and q-arms. Finally, by arranging into a descending order according to the obtained measured masses, the X-ray karyotypes of both the B and T lymphocytes cells are displayed in Fig. 5.

In placing the chromosomes in sequence, we took note of the positions of the sex chromosomes: the X chromosome was placed between chromosomes 7 and 8, while the Y chromosome was placed between chromosomes 19 and 22. The Yoruba B cell line (Fig. 5a) was male (XY) while the lymphocyte T cell (Fig. 5b) was female (XX). In addition, because of errors in the original assignments, chromosome 11 falls before chromosome 10; similarly, chromosome 20 falls before chromosome 19 and chromosome 22 before chromosome 21. Chromosome 1 has the highest mass and chromosome 21 has the lowest mass.

Currently, chromosomes are karyotyped on the basis of morphology, volume and the DNA content using techniques such as M-FISH (Yusuf et al., 2011) (Anderson et al., 2002), Serial Block-face Scanning Electron Microscopy (SBFSEM) (Chen et al., 2017) and flow cytometry (Harris et al., 1986). We can reliably karyotype the chromosomes from group A (chromosomes 1–3) and B (chromosomes 4 and 5), similar to the conventional methods of chromosome karyotypes. However, for the smaller chromosomes, we observe shuffling by visualising their morphology in Fig. 5. In the future, correlative imaging may be performed by M-FISH on fixed human metaphase chromosomes (Shemilt et al., 2015), followed by coherent X-ray scanning ptychography on the same spread to independently verify the X-ray karyotype of chromosomes.

For further quantitative comparison with the known number of DNA base pairs for each chromosome in the human genome sequence, we produced graphical X-ray karyotypes of each spread, plotting the measured mass against the number of base pairs. These are discussed in the following two sections, reporting the results of our staining tests of B cells in the first experiment and the irradiated T cells in the second experiment.

### Masses of stained B cell chromosomes

Investigation of biological samples with electron microscopy and hard X-ray imaging is very challenging because of the weak scattering of its light atoms. We therefore investigated staining the chromosomes with 1% uranyl acetate and 6 mM platinum blue in an attempt to increase the phase contrast (see the ‘Materials and methods’ section). Staining deposits a thin layer of additional electron density on the top of the chromosome samples to increase the scattering intensity of the beam. Chromosome-specific staining could also help to visualise and segment the chromosomes and separate them from nuclei and other debris.

**Fig. 6:**
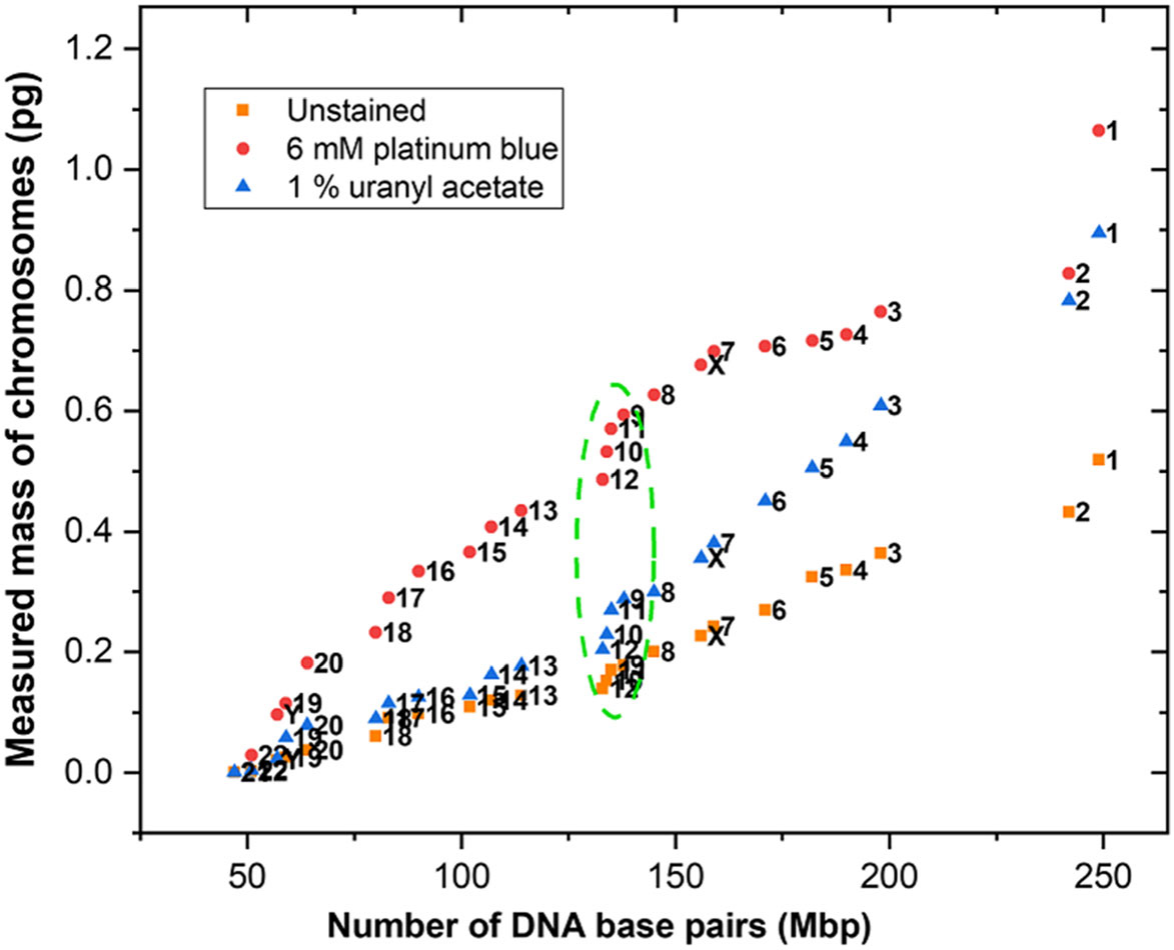
The X-ray karyotype compares the measured masses of individual chromosomes, stained with heavy metals and unstained. The masses were retrieved from the ptychography phase images of B lymphocyte cells. The green dashed oval encircles the region of chromosomes 9–12, from each spread

The X-ray phase ptychography images were segmented with the ‘free hand tool’ in ImageJ to obtain the raw integer density (RawIntDen) value. The average background pixel values were subtracted and the electron count values were obtained using Eq. (1). The number of electrons present in each individual chromosome was multiplied by the mass of the two nucleons (2 Da = 3.35×10^−27^ kg) to generate a table of the observed masses of each individual chromosome. Graphs are plotted in Fig. 6 of the ranked masses *versus* the known number of DNA base pairs of each individual chromosome to produce an X-ray karyotype from each spread. As with other karyotype methodologies, we assume the sequence of observed masses follows the same numbering of the chromosomes.

The unstained chromosome 1s have masses of 0.55 pg and 0.48 pg, compared with 0.87 pg estimated above for a single chromatid. An additional factor of 2 is expected because two genome copies present in the chromosomes at metaphase. The measurements therefore fall well short of accounting for the known DNA and protein components of the structure. As discussed above, we think this is explained by a mass loss due to long-term storage of the prepared chromosomes before imaging. Because the same storage conditions were applied to the stained samples, our report on the effects of staining is still valid and interesting.

In Fig. 6, it is clearly seen that stained chromosomes are approximately 2–3 times heavier in their measured masses, relative to the unstained example, with some notable differences in the magnitude of the staining effect on different chromosomes. We can conclude that the heavy atom staining can add on up to 100% extra mass to the stained chromosomes. There are differential staining effects visible between the UA and Pt-blue agents. The heaviest chromosomes are stained almost equally, while the intermediate masses, particularly the tightly bunched region of chromosomes 9–12 (green dashed oval in Fig. 6), show differences close to a factor of three. The lower-mass chromosomes are almost unaffected by UA, while they are preferentially stained by Pt-blue. It is likely that differential staining may have affected the numerical identification of chromosomes based on ranking of their masses, but we note the clustering of chromosomes 9–12 (green dashed oval in Fig. 6) is preserved by the staining in both cases.

We note the mass measurements slightly underestimate the mass of the stain because the heavy elements (U and Pt) contain additional neutrons to the simple 1:1 proton:neutron ratio of light elements. The potential benefit of heavy metal stain is to improve the contrast of the weak scattering biological sample and this has been observed for both the stains used. We note also that we missed one chromosome in UA stained and two chromosomes in the platinum blue stained spread. The sum of the measured masses (45 out of 46 chromosomes) is 13.2 pg for UA stain and 22.2 pg for Pt-blue stain (44 out of 46), compared with 8.3 pg for the unstained sample (46 out of 46).

Uranyl acetate (UA) and platinum blue stains both enhance the phase contrast of electron density maps due to increased scattering (Cao et al., 2011) (De Carlo & Harris, 2012) (Yusuf et al., 2014b, c). UA is radioactive and known to be toxic to the biological specimens. Relevant to our results is the claim that it disturbs the protein-protein/DNA-protein interactions and induces conformational changes (Lin, 2020); this may explain the bigger distortion seen in the shape of the UA X-ray karyotype in Fig. 6. Moreover, UA can form microcrystals once dried on the substrate (De Carlo & Harris, 2012). Consequently, the less toxic platinum blue stain is a preferred substitute for UA in TEM and SEM imaging (Inaga et al., 2007; Wanner & Formanek, 1995).

In the X-ray mass karyotypes from both stained and unstained chromosome spreads, it is observed that the larger chromosomes 1–2 have the highest variability among their homologues, followed by chromosomes 3–6, while for smaller chromosomes the variability of base pair ratio decreases (Korenberg & Engels, 1978). Moreover, the base ratios (AT *vs* GC) also vary between the segments of a chromosome and among full sets of chromosomes (Korenberg & Engels, 1978). The other quantitative karyotyping techniques, including bivariate flow cytometry approaches, do not cleanly resolve chromosomes 9–12, perhaps because of their similar DNA content and the base pair compositions (Mendelsohn et al., 1973) (Korenberg & Engels, 1978) (Langlois et al., 1982) (Boschman et al., 1991). Two-colour flow cytometry with different dyes allows separation of these chromosomes.

The phase contrast imaging technique is adequately sensitive to resolve the relative masses of chromosomes 9–12 in linear regression karyotypes, as this method gives the total mass of DNA content and chromosomal proteins present. This could be a consequence of the significant difference in their masses and can be easily identified in a linear regression. The green oval in Fig. 6 indicates the concentrated data points of chromosomes 9–12, where the measured masses are well-separated in contrast to their similar genome lengths.

In the second round of experiments, discussed next, following improvements of the reconstruction algorithms (Batey, 2014) and the use of more freshly prepared samples, we had enough sensitivity to avoid the need for of staining and performed the whole radiation dose experiment with unstained chromosome spreads. The quantitative conclusions drawn below required the use of unmodified mass data.

### Masses of irradiated T cell chromosomes

**Fig. 7:**
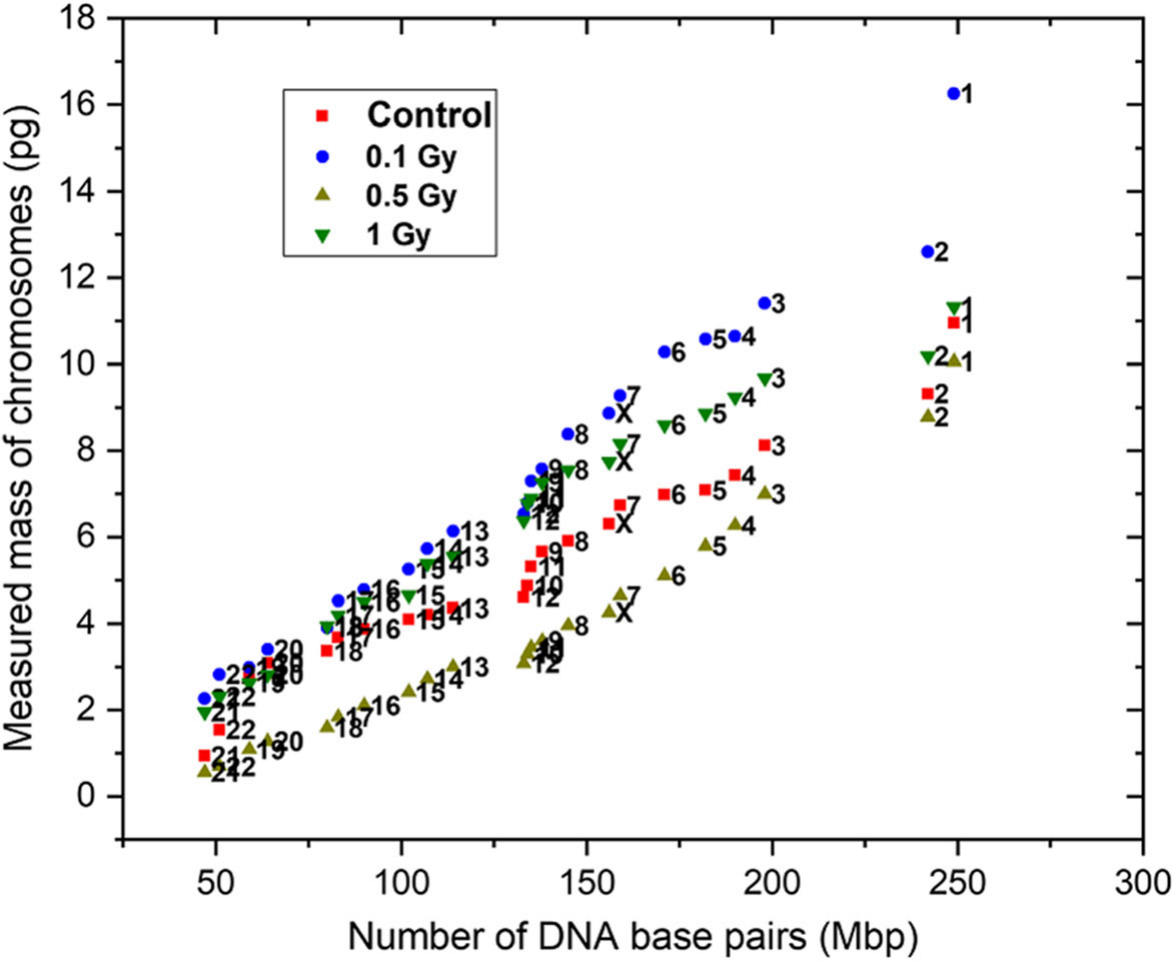
X-ray karyotype of the average mass of homologous pairs of chromosomes segmented from the phase image of the spreads obtained from T lymphocyte cells shown in Fig. 5. Graphs compare unstained chromosomes (‘control’) with irradiated chromosomes at three dose levels indicated

The outcome of the irradiation effects in live T lymphocyte cells is shown in Fig. 7. The data points fall on a straight line, indicating there is a linear relationship between measured masses and the known number of DNA base pairs. The total masses, summed over all 46 chromosomes, were 242.2 pg, for the unstained sample (‘control’) and 336.5 pg, 172.9 pg and 293.1 pg, for the radiation doses, 0.1 Gy, 0.5 Gy and 1 Gy respectively. Surprisingly, the total mass for 0.1 Gy is higher than the non-irradiated chromosomes, then significantly decreases for 0.5 Gy before rising again for 1 Gy.

The average mass of chromosome 1 is now 10.9 pg for the un-irradiated spread, compared with 0.52 pg seen in the first experiment and 1.74 pg estimate from the genome size and known protein complement. This six-fold excess mass could indicate a gross underestimation of the associated protein component, or that there are other molecules originating from the cytoplasm during the preparation, notably from the nuclear membrane, and also water of solvation and ions. Moreover, it is seen from each X-ray karyotype plot that the average mass of the two chromosome 1s is much higher than the expected extrapolation from the rest of the chromosomes in the spread, irrespective of radiation doses.

Ionising radiation effects on the mass content of metaphase chromosome were investigated by the X-ray ptychography technique. In the course of a typical human cell cycle, before, the DNA synthesis in S-phase, the cell nucleus will consist of 46 chromatids and after that it will replicate to 92 chromatids prior to separation at anaphase to form two daughter cells. Beyond the conserved histones, which form stoichiometric DNA-protein complexes (Maeshima & Eltsov, 2008), the non-histone protein content is expected to differ at different stages of the cell cycle (Gookin et al., 2017). For example, protein from the ‘minichromosome maintenance’ (MCM) family binds to the replicating point of the DNA at the G1-phase and then disappears once the replication starts (Forsburg, 2004). Similarly, condensin and topoisomerase IIα proteins, involved in the condensation and decatenation of chromosomes, appear at the S-phase after DNA replication and then disintegrate once the chromosomes are segregated properly at anaphase (Charbin et al., 2014). Overall, 209 interphase proteins and 107 metaphase proteins have been identified in human cells, with assorted localisation and function (Uchiyama et al., 2005).

Figure 7 indicates the effect of ionising radiation on the acquired measured masses of a full set of chromosomes. One hypothesis is that we should expect a decreasing trend in mass with increased induced X-ray irradiation doses, possibly due to varied DNA lesions (Borrego-Soto et al., 2015) and protein modifications upon X-ray doses (Reisz et al., 2014) (Lowe et al., 2020). With 0.1 Gy and 1 Gy, the total obtained quantitative masses were 336.5 pg and 293.1 pg, a noticeable increase in mass relative to the non-irradiated chromosome spread with 242.2 pg. At 0.5-Gy dose, the measured mass decreased to 172.9 pg.

To understand these results, we consider that, upon irradiation, various stress responsive enzymes and proteins are recruited, including DNA-PKs, 53BP1 and Ku70/Ku80 (Biau et al., 2019). DNA damage response signalling pathway activates the NHEJ and HRR mechanism to repair the DNA lesions (Mahaney et al., 2009). Immediately after radiation exposure, the cell cycle checkpoint response, with its related proteins, also comes into play (Mahaney et al., 2009). It has been observed in male germ-line cells that with increased doses of ionising radiation, the double-strand break (DSB) number increases. On the other hand, the repair mechanism activates and repairs the lesions within 4 to 16 h (Singh et al., 2018). Therefore, we can postulate that the extra mass may be added to the irradiated chromosome spreads which could be because of the proteins and enzymes coming into play to initiate the repair mechanism of the various DNA lesions that occurred immediately after the irradiation at 0.1 Gy dose. However, this does not explain the lower masses found at 0.5 Gy dose which, interestingly, has relatively low mass compared to non-irradiated chromosomes and other chosen X-ray doses. Interestingly, the study of Neumaier et al. (2012) showed that ‘Radiation-induced foci’ (RIF) were more at 0.1 Gy (64 RIF/Gy) dose compared to 1 Gy (23 RIF/Gy) dose, which may mean the number of DSBs is independent of irradiation doses in human cells at low dose levels. Apparently, relative DNA repair proteins starts the repair mechanism after exposure.

Furthermore, in the literature, the radiosensitivity of immune cells has been studied for low-dose radiotherapy (LDRT) and high-dose radiotherapy (HDRT). It has been shown that the viability of the cells decreases after 1 Gy exposure and that aberration increases, leading to apoptosis and cell death at doses above 10 Gy (Falcke et al., 2018). Continuous exposure to ionising radiation leads to histone protein degradation up to 40%, after irradiating with dose rates of 6 mGy/h to 20 mGy/h for 7 days (Lowe et al., 2020).

### Effect of radiation exposure during imaging

**Fig. 8:**
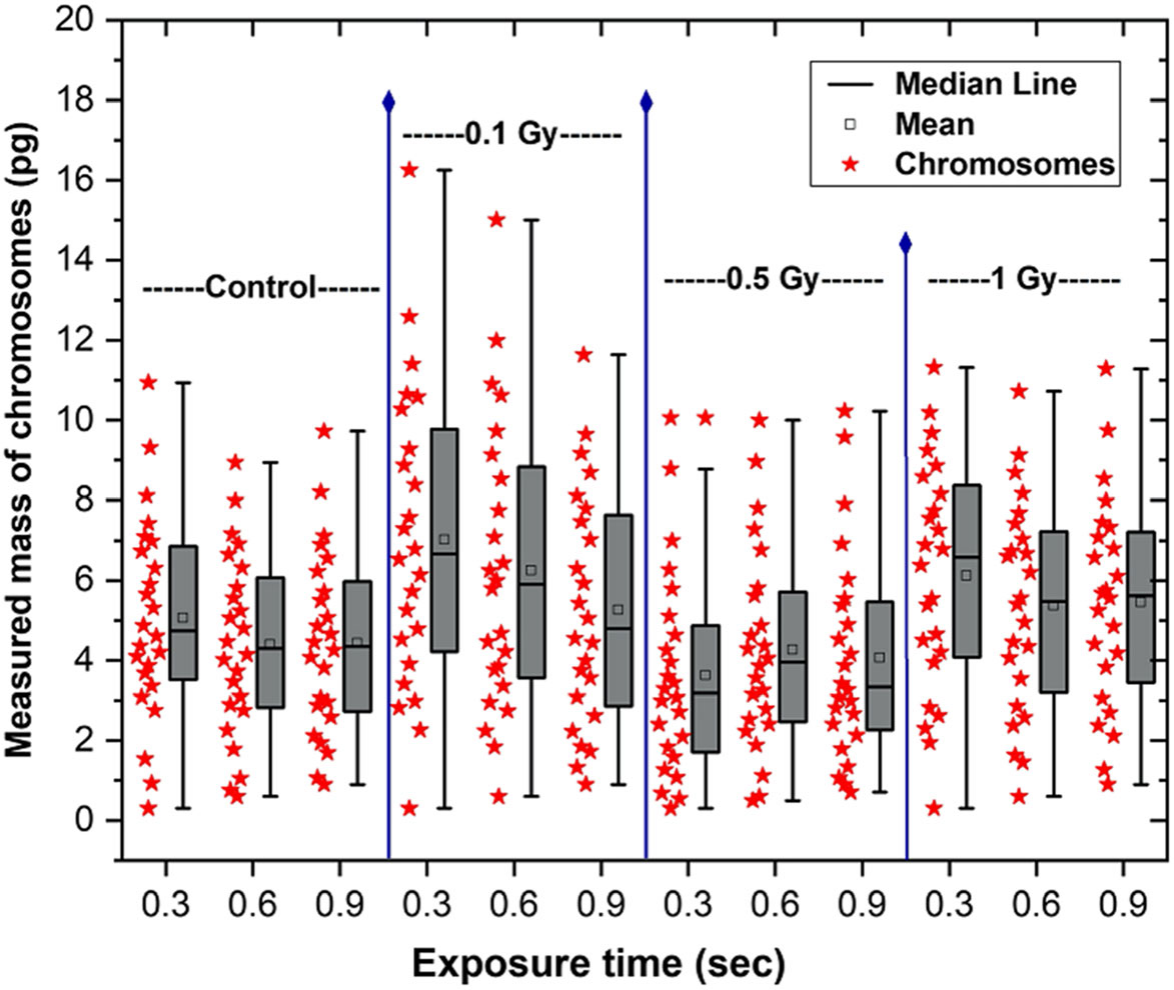
Measured masses of chromosomes from reconstructed phase images of non-irradiated and irradiated chromosomes spreads. The *x*-axis represents the X-ray exposure time given to each sample during imaging. The red stars show the averaged masses of homologous pairs (22, XX) chromosomes from the obtained spreads of each sample. The chromosomes are in sequence from chromosome 1 at the top to chromosome 21 at the bottom. The boxes alongside of red stars represents the maximum and minimum values of each spread. The mean and the median lines are also shown inside the boxes

As a follow-up investigation, we measured the quantitative masses for different exposure times used in the X-ray ptychography measurement. We note that the measurement doses are in the MGy range and far exceed those given to the live cells in culture; however, since the material is fixed before imaging, there will not be any cellular irradiation response expected. The same methanol: acetic acid (3:1) fixed human metaphase chromosome spread obtained from primary T lymphocytes was scanned for three different image exposure times, 0.3 s, 0.6 s and 0.9 s in sequence. We collected the diffraction patterns from both irradiated (0.1 Gy, 0.5 Gy and 1 Gy) and non-irradiated (control) chromosomes. In Fig. 8, the masses of 22 pairs of autosomes and one pair of sex chromosomes (XX) are reported following the same data analysis stream described above. The stated mass of each chromosome is the averaged mass of two homologous chromosomes.

The plot in Fig. 8 depicts the following information for each exposure time: the recorded chromosome masses are arrayed as star-shaped data points; the rectangular shaded box shows the distribution of 50% of the mass data points, the upper and lower whiskers defines the maxima and minima, respectively, and the horizontal line and the small box inside the big box represent the median and the average value of each distribution, respectively.

For the non-irradiated (control) sample, the summed masses (of all 46 chromosomes at metaphase stage) were as follows: 242.2 pg, 209.7 pg and 210.8 pg for 0.3 s, 0.6 s and 0.9 s, respectively. Longer exposure to ionising radiation eventually leads to destruction of the biological samples (Lowe et al., 2020; Reisz et al., 2014) especially while measuring at room temperature without any cryoprotectant. Cryo-imaging is the future of the biological imaging (Yusuf et al., 2017) (Yusuf et al., 2019).

A decrease with exposure sequence was observed in the measured masses which was similarly observed for 0.1 Gy and 1 Gy irradiations. The summed masses for 0.1 Gy irradiation were 336.5 pg, 298. 7 pg and 250.7 pg at 0.3 s, 0.6 s and 0.9 s, while at 1 Gy they were 293.1 pg, 256.5 pg and 259.7 pg, respectively. However, the summed masses at 0.5 Gy dose were 172.9 pg, 203.4 pg and 193.4 pg at 0.3 s, 0.6 s and 0.9 s respectively, suggesting an increasing trend. It may be that the results of Fig. 8 mainly reflect the overall reproducibility of the mass-determination experiment, at the level of about 10%, without any significant effect of exposure time over the range studied. However, the increase in measured masses observed at 0.1 Gy and 1 Gy relative to the non-irradiated control sample is statistically significant at all exposure times, and supports our hypothesis of the immediate initiation of cell cycle checkpoint responses and the activation of DNA damage repair protein signalling pathway, which add mass to the irradiated chromosomes (Huang & Zhou, 2020).

## Conclusions and outlook

The observation is that using ptychography, the mass of the chromosomes is seen to increase upon irradiation at 0.1 Gy compared to 0.5 Gy or higher is unusual. However, we speculate that since the adaptive response to lowdose irradiation (< 0.1 Gy) or low dose rate (0.06 mSv/h) has been reported to increase certain cell proliferation (von Sallmann, 1952), a similar process may apply to DNA induction but this is as yet untested or unknown. It is worth noting that in a separate technique using fluorescence lifetime imaging microscopy, we show that there is a correlation between 0.1 Gy radiation–induced excited state lifetime decrease over 0.5 Gy and non-radiation of cell chromosomes (Bhartiya, 2021), thus indicating an effect on chromosomes following ionising radiation at 0.1 Gy. Low-dose irradiation is thought to induce an adaptive or hormetic response under certain conditions (Luckey, 2006; van Wyngaarden & Pauwels, 1995). Further studies are needed to fully characterise these effects.

Our work demonstrates the ability to extract the individual masses of complete metaphase human chromosome spreads using two-dimensional phase contrast images to construct an X-ray karyotype. The successful combination of scanning X-ray ptychography technologies allows us to measure the masses of stained and unstained metaphase human chromosomes at room temperature and gives a good correlation between known genome sequence and the measured mass of the chromosomes obtained using X-ray ptychography. This technique is useful for determining the total genome mass of a species, including DNA and the related chromosomal proteins present at the different stages of the cell cycle without invasive staining.

The obtained quantitative information allowed us to measure the masses from uranyl acetate and platinum-blue-stained chromosome spreads obtained from B lymphocytes and build an X-ray karyotype. We observed a significant mass gain associated with heavy-metal staining. The same procedure was applied to extract the masses from unstained X-ray-induced chromosome spreads obtained from T lymphocytes. The X-ray karyotype was also generated from chromosome spreads with different exposure during X-ray scanning giving consistent results.

The individual measured masses of isolated chromosomes provide information of their DNA content along with the relative chromosomal proteins divided into histones and non-histones. Henceforth, measured and expected masses of each individual chromosome can be determined. Furthermore, with X-ray ptychography, the difference in masses of all 46 human chromosomes can be visualised in a linear regression plot, including chromosomes 9–12, which is not possible in two-colour flow cytometry, because of their very similar DNA content.

## Abbreviations

CA: Chromosomal aberration
CDI: Coherent diffraction imaging
CS: Central stop
DSB: Double strand break
ePIE: Extended Ptychographical Iterative Engine
FBS: Fetal bovine serum
FoV: Field of view
FZP: Fresnel zone plate
HBSS: Hank’s balanced salt solution
HDRT: High-dose radiotherapy
HIO: Hybrid input output
IR: Ionising radiation
LET: Linear energy transfer
LDRT: Low-dose radiotherapy
MAA: Methanol acetic acid
MCM: Minichromosome maintenance
M-FISH: Multiplex fluorescence in situ hybridization
OSA: Order sorting aperature
PDB: Protein data bank
PHA: Phytohaemagglutinin
PHE: Public health England
PIE: Ptychographical iterative engine
RIF: Radiation-induced foci
SXT: Soft X-ray tomography
TEM: Transmission electron microscopy
UA: Uranyl acetate
XFM: X-ray fluorescence microscopy

## References

[CR1] AbdulSalam SF, Thowfeik FS, Merino EJ (2017). Excessive reactive oxygen species and exotic DNA lesions as an exploitable liability. Biochemistry.

[CR2] Als-Nielsen J, McMorrow D (2011) Elements of modern X-ray physics, second edn. Wiley. 10.1002/9781119998365

[CR3] Anderson R (2010) Multiplex Fluorescence in situ Hybridization (M-FISH). In: Bridger JM, Volpi EV (eds) Fluorescence in situ Hybridization (FISH) Protocols and Applications. Humana Press, pp 83–97

[CR4] Anderson RM, Stevens DL, Goodhead DT (2002). M-FISH analysis shows that complex chromosome aberrations induced by alpha-particle tracks are cumulative products of localized rearrangements. PNAS.

[CR5] Azzam E, Jay-Gerin J, Pain D (2014). Ionizing radiation-induced metabolic oxidative stress and prolonged cell injury. Cancer Letters.

[CR6] Balajee AS, Bertucci A, Taveras M, Brenner DJ (2014). Multicolour FISH analysis of ionising radiation induced micronucleus formation in human lymphocytes. Mutagenesis.

[CR7] Batey, D. J. (2014) Ptychographic imaging of mixed states. University of Sheffield. Available at: http://etheses.whiterose.ac.uk/8524/.

[CR8] Beckers M, Senkbeil T, Gorniak T, Reese M, Giewekemeyer K, Gleber SC, Salditt T, Rosenhahn A (2011). Chemical contrast in soft X-ray ptychography. Physical Review Letters.

[CR9] Bench GS, Friz AM, Corzett MH, Morse DH, Balhorn R (1996). DNA and total protamine masses in individual sperm from fertile mammalian subjects. Cytometry.

[CR10] Bhartiya, A. (2021) ‘Radiation effects on the structure of chromosomes’, PhD Thesis, University College London

[CR11] Biau J, Chautard E, Verrelle P, Dutreix M (2019). Altering DNA repair to improve radiation therapy: specific and multiple pathway targeting. Frontiers in Oncology.

[CR12] Borrego-Soto G, Ortiz-López R, Rojas-Martínez A (2015). Ionizing radiation-induced DNA injury and damage detection in patients with breast cancer. Genetics and Molecular Biology.

[CR13] Boschman GA, Rens W, van Oven CH, Manders EMM, Aten JA (1991). Bivariate flow karyotyping of human chromosomes: evaluation of variation in Hoechst 33258 fluorescence, chromomycin A3 fluorescence, and relative chromosomal DNA content. Cytometry.

[CR14] Botchway SW, Stevens DL, Hill MA, Jenner TJ, O'Neill P (1997). Induction and rejoining of DNA double-strand breaks in Chinese hamster V79-4 cells irradiated with characteristic aluminum K and copper L ultrasoft X rays. Radiation Research.

[CR15] Brown TA (2002). Genomes. second. Oxford.

[CR16] Cao B, Xu H, Mao C (2011). Transmission electron microscopy as a tool to image bioinorganic nanohybrids: the case of phage-gold nanocomposites. Microscopy Research and Technique.

[CR17] Chapman HN, Nugent KA (2010). Coherent lensless X-ray imaging. Nature Photonics, Nature Publishing Group.

[CR18] Chapman HN, Barty A, Marchesini S, Noy A, Cui C, Howells M, Rosen R, He H, Spence JC, Beetz T, Jacobsen C, Shapiro D, Hau-Riege SP (2006). High-resolution ab initio three-dimensional x-ray diffraction microscopy. Journal of the Optical Society of America. A.

[CR19] Charbin A, Bouchoux C, Uhlmann F (2014). Condensin aids sister chromatid decatenation by topoisomerase II. Nucleic Acids Research.

[CR20] Chen B, Yusuf M, Hashimoto T, Estandarte AK, Thompson G, Robinson I (2017). Three-dimensional positioning and structure of chromosomes in a human prophase nucleus. Science Advances.

[CR21] Cram LS, Bartholdi MF, Ray FA, Meyne J, Moyzis RK, Schwarzacher-Robinson T, Kraemer PM (1988). Overview of flow cytogenetics for clinical applications. Cytometry.

[CR22] De Carlo S, Harris JR (2012). Negative staining and cryo-negative staining of macromolecules and viruses for TEM. Micron.

[CR23] Deng J, Vine DJ, Chen S, Nashed YSG, Jin Q, Peterka T, Vogt S, Jacobsen C (2015). Advances and challenges in cryo ptychography at the Advanced Photon Source. PNAS.

[CR24] Deng J, Chen S, Jin Q, Vacek E, Jacobsen C, Lai B, Vogt S (2019). Correlative X-ray ptychographic and fluorescence imaging at the Advanced Photon Source. Microscopy and Microanalysis.

[CR25] Desouky O, Ding N, Zhou G (2015). ScienceDirect targeted and non-targeted effects of ionizing radiation. Journal of Radiation Research and Applied Science.

[CR26] Dierolf M, Menzel A, Thibault P, Schneider P, Kewish CM, Wepf R, Bunk O, Pfeiffer F (2010). Ptychographic X-ray computed tomography at the nanoscale. Nature.

[CR27] Doležel J, Bartoš J, Voglmayr H, Greilhuber J (2003). Nuclear DNA content and genome size of trout and human. Cytometry Part A.

[CR28] Durante M, Formenti SC (2018). Radiation-induced chromosomal aberrations and immunotherapy: micronuclei, cytosolic DNA, and interferon-production pathway. Frontiers in Oncology.

[CR29] Estandarte AK, Botchway S, Lynch C, Yusuf M, Robinson I (2016) The use of DAPI fluorescence lifetime imaging for investigating chromatin condensation in human chromosomes. Scientific Reports 6:1–12. 10.1038/srep31417 10.1038/srep31417 PMC4985626 27526631

[CR30] Falcke SE, Rühle PF, Deloch L, Fietkau R, Frey B, Gaipl US (2018). Clinically relevant radiation exposure differentially impacts forms of cell death in human cells of the innate and adaptive immune system. International Journal of Molecular Sciences.

[CR31] Forsburg SL (2004). Eukaryotic MCM proteins : beyond replication initiation. American Society for Microbiology.

[CR32] Fujimichi Y, Hamada N (2014). Ionizing irradiation not only inactivates clonogenic potential in primary normal human diploid lens epithelial cells but also stimulates cell proliferation in a subset of this population. PLoS One.

[CR33] Goldfeder RL, Wall DP, Khoury MJ, Ioannidis JPA, Ashley EA (2017). Human genome sequencing at the population scale: a primer on high-throughput DNA sequencing and analysis. American Journal of Epidemiology.

[CR34] Gookin S, Min M, Phadke H, Chung M, Moser J, Miller I, Carter D, Spencer SL (2017). A map of protein dynamics during cell-cycle progression and cell-cycle exit’. Public Library of Science: Biology.

[CR35] Grimwade D, Hills RK, Moorman AV, Walker H, Chatters S, Goldstone AH, Wheatley K, Harrison CJ, Burnett AK (2010). Refinement of cytogenetic classification in acute myeloid leukemia: determination of prognostic significance of rare recurring chromosomal abnormalities among 5876 younger adult patients treated in the United Kingdom Medical Research Council trials. Blood.

[CR36] Guk BK, Yae JY, Tae JS, Hwa SY, Yong SG, Sang JL (2008). X-ray imaging of various biological samples using a phase-contrast hard X-ray microscope. Microscopy Research and Technique.

[CR37] Gulston M, de Lara C, Jenner T, Davis E, O'Neill P (2004). Processing of clustered DNA damage generates additional double-strand breaks in mammalian cells post-irradiation. Nucleic Acids Research.

[CR38] Harris P, Boyd E, Young BD, Ferguson-Smith MA (1986). Determination of the DNA content of human chromosomes by flow cytometry. Cytogenetics and Cell Genetics.

[CR39] Harshman SW, Young NL, Parthun MR, Freitas MA (2013). H1 histones: current perspectives and challenges. Nucleic Acids Research.

[CR40] Hémonnot CYJ, Köster S (2017). Imaging of biological materials and cells by X-ray scattering and diffraction. Nanotechnology.

[CR41] Howells M, Jacobsen C, Warwick T, Van den Bos A (2006) Principles and applications of zone plate X-ray microscopes. In: Hawkes PW, Spence JCH (eds) Science of Microscopy. Springer, pp 835–926. 10.1016/s1369-7021(06)71581-4

[CR42] Huang RX, Zhou PK (2020). DNA damage response signaling pathways and targets for radiotherapy sensitization in cancer. Signal Transduction and Targeted Therapy.

[CR43] Huang X, Nelson J, Kirz J, Lima E, Marchesini S, Miao H, Neiman AM, Shapiro D, Steinbrener J, Stewart A, Turner JJ, Jacobsen C (2009). Soft X-ray diffraction microscopy of a frozen hydrated yeast cell. Physical Review Letters.

[CR44] Inaga S, Katsumoto T, Tanaka K, Kameie T, Nakane H, Naguro T (2007) Platinum blue as an alternative to uranyl acetate for staining in transmission electron microscopy. Archives of Histology and Cytology:43–49. 10.1679/aohc.70.43 10.1679/aohc.70.43 17558143

[CR45] Jain AK et al (2017) Chromosomal aberrations. In: Kumar A et al (eds) Mutagenicity: Assays and Applications, 1st edn. Academic Press, pp 69–83

[CR46] Kirz J, Jacobsen C, Howells M (1995). Soft X-ray microscopes and their biological applications. Quarterly Reviews of Biophysics.

[CR47] Korenberg JR, Engels WR (1978). Base ratio, DNA content, and quinacrine-brightness of human chromosomes. PNAS.

[CR48] Lander ES (2001). Initial sequencing and analysis of the human genome. Nature.

[CR49] Langlois RG, Yu LC, Gray JW, Carrano AV (1982). Quantitative karyotyping of human chromosomes by dual beam flow cytometry. PNAS.

[CR50] Larabell CA, Nugent KA (2010). Imaging cellular architecture with X-rays. Current Opinion in Structural Biology.

[CR51] Lin YW (2020). Uranyl binding to proteins and structural-functional impacts. Biomolecules.

[CR52] Lomax ME, Folkes LK, Neill PO (2013). Biological consequences of radiation-induced DNA damage : relevance to radiotherapy. Clinical Oncology.

[CR53] Lowe DJ, Herzog M, Mosler T, Cohen H, Felton S, Beli P, Raj K, Galanty Y, Jackson SP (2020). Chronic irradiation of human cells reduces histone levels and deregulates gene expression. Scientific Reports.

[CR54] Luckey TD (2006). Radiation hormesis: the good, the bad, and the ugly. Dose-Response.

[CR55] Maeshima K, Eltsov M (2008). Packaging the genome: the structure of mitotic chromosomes. Journal of Biochemistry.

[CR56] Maeshima K, Imai R, Tamura S, Nozaki T (2014). Chromatin as dynamic 10-nm fibers. Chromosoma.

[CR57] Magnander K, Hultborn R, Claesson K, Elmroth K (2010). Clustered DNA damage in irradiated human diploid fibroblasts: influence of chromatin organization. Radiation Research.

[CR58] Mahaney BL, Meek K, Lees-Miller SP (2009). Repair of ionizing radiation-induced DNA double strand breaks by non-homologous end-joining. Biochemistry.

[CR59] Maiden AM, Rodenburg JM (2009). An improved ptychographical phase retrieval algorithm for diffractive imaging. Ultramicroscopy.

[CR60] Maiden A, Johnson D, Li P (2017). Further improvements to the ptychographical iterative engine. Optica.

[CR61] Maser J, Osanna A, Wang Y, Jacobsen C, Kirz J, Spector S, Winn B, Tennant D (2000). Soft X-ray microscopy with a cryo scanning transmission X-ray microscope : I . Instrumentation, imaging and spectroscopy. Journal of Microscopy.

[CR62] Mendelsohn ML, Mayall BH, Bogart E, Moore DH, Perry BH (1973). DNA content and DNA-based centromeric Index of the 24 human chromosomes. Science.

[CR63] Miao J, Sayre D, Chapman HN (1998) Phase retrieval from the magnitude of the Fourier transforms of nonperiodic objects. Journal of the Optical Society of America A 15:1662–1669. 10.1364/JOSAA.15.001662

[CR64] Moralli D, Yusuf M, Mandegar MA, Khoja S, Monaco ZL, Volpi EV (2011). An improved technique for chromosomal analysis of human ES and iPS cells. Stem Cell Reviews and Reports.

[CR65] Nakano T, Xu X, Salem AMH, Shoulkamy MI, Ide H (2017). Radiation-induced DNA–protein cross-links: mechanisms and biological significance. Free Radical Biology and Medicine.

[CR66] Neumaier T, Swenson J, Pham C, Polyzos A, Lo AT, Yang PA, Dyball J, Asaithamby A, Chen DJ, Bissell MJ, Thalhammer S, Costes SV (2012). Evidence for formation of DNA repair centers and dose-response nonlinearity in human cells. PNAS.

[CR67] Nishino Y, Takahashi Y, Imamoto N, Ishikawa T, Maeshima K (2009). Three-dimensional visualization of a human chromosome using coherent X-ray diffraction. Physical Review Letters.

[CR68] Olins A L, Olins DE (1974) Spheroid chromatin units (ν Bodies). Science 183:330–332. 10.1126/science.183.4122.330 10.1126/science.183.4122.330 4128918

[CR69] Piovesan A, Pelleri MC, Antonaros F, Strippoli P, Caracausi M, Vitale L (2019). On the length, weight and GC content of the human genome. BMC Research Notes.

[CR70] Reisz JA, Bansal N, Qian J, Zhao W, Furdui CM (2014). Effects of ionizing radiation on biological molecules - mechanisms of damage and emerging methods of detection. Antioxidants and Redox Signaling.

[CR71] Ried T, Schröck E, Ning Y, Wienberg J (1998). Chromosome painting: a useful art. Human Molecular Genetics.

[CR72] Robinson I, Yusuf M, Schwenke J, Estandarte A, Zhang F, Bhella G, Parmar N, Clark J, Song C, Nam D, Ratnasari G, Kaneyoshi K, Takata H, Fukui K (2015). Damage-free imaging of human chromosomes’. Chromosome Science.

[CR73] Robinson I, Yang Y, Zhang F, Lynch C, Yusuf M, Cloetens P (2016). Nuclear incorporation of iron during the eukaryotic cell cycle. Journal of Synchrotron Radiation.

[CR74] Rodenburg JM, Hurst AC, Cullis AG, Dobson BR, Pfeiffer F, Bunk O, David C, Jefimovs K, Johnson I (2007). Hard-X-ray lensless imaging of extended objects. Physical Review Letters.

[CR75] Shapiro D, Thibault P, Beetz T, Elser V, Howells M, Jacobsen C, Kirz J, Lima E, Miao H, Neiman AM, Sayre D (2005) Biological imaging by soft x-ray diffraction microscopy. Proceedings of the National Academy of Sciences of the United States of America 102:15343–15346. 10.1073/pnas.0503305102 10.1073/pnas.0503305102 PMC1250270 16219701

[CR76] Shemilt L, Verbanis E, Schwenke J, Estandarte AK, Xiong G, Harder R, Parmar N, Yusuf M, Zhang F, Robinson IK (2015). Karyotyping human chromosomes by optical and x-ray ptychography methods. Biophysical Journal.

[CR77] Singh P, Aggarwal LM, Parry SA, Raman MJ (2018). Radiation dosimetry and repair kinetics of DNA damage foci in mouse pachytene spermatocyte and round spermatid stages. Mutagenesis.

[CR78] Smith EA, McDermott G, Do M, Leung K, Panning B, Le Gros MA, Larabell CA (2014). Quantitatively imaging chromosomes by correlated cryo-fluorescence and soft X-ray tomographies. Biophysical Journal.

[CR79] Speicher MR, Ballard SG, Ward DC (1996) Karyotyping human chromosomes by combinatorial multi-fluor FISH. Nature Genetics 12:368–375 10.1038/ng0496-368 8630489

[CR80] Strachan T, Read AP (2004) Chromosome structure and function, in Human Molecular Genetics, 3rd edn. Garland Science: Taylor & Francis Group, London and New York, pp 34–36

[CR81] Travers A (2014). Structural biology. The 30-nm fiber redux. Science.

[CR82] Tobias E (2011) Chromosome aberrations. In: Essential Medical Genetics, 6th edn. Wiley-Blackwell, pp 89–111

[CR83] Touil N, Elhajouji A, Thierens H, Kirsch-Volders M (2000). Analysis of chromosome loss and chromosome segregation in cytokinesis-blocked human lymphocytes: non-disjunction is the prevalent mistake in chromosome segregation produced by low dose exposure to ionizing radiation. Mutagenesis.

[CR84] Tremethick DJ (2007). Higher-order structures of chromatin: the Elusive 30 nm Fiber David. Cell.

[CR85] Uchiyama S, Kobayashi S, Takata H, Ishihara T, Hori N, Higashi T, Hayashihara K, Sone T, Higo D, Nirasawa T, Takao T, Matsunaga S, Fukui K (2005). Proteome analysis of human metaphase chromosomes. Journal of Biological Chemistry.

[CR86] van Wyngaarden KE, Pauwels EKJ (1995). Hormesis: are low doses of ionizing radiation harmful or beneficial?. European Journal of Nuclear Medicine.

[CR87] von Sallmann L (1952). Experimental studies on early lens changes after roentgen irradiation. III. Effect of x-radiation on mitotic activity and nuclear fragmentation of lens epithelium in normal and cysteine-treated rabbits. A.M.A. Archives of Ophthalmology.

[CR88] Wanner G, Formanek H (1995). Imaging of DNA in human and plant chromosomes by high-resolution scanning electron microscopy. Chromosome Research.

[CR89] Yamamoto Y, Shinohara K (2002). Application of X-ray microscopy in analysis of living hydrated cells. Anatomical Record.

[CR90] Yan H, Nazaretski E, Lauer K, Huang X, Wagner U, Rau C, Yusuf M, Robinson I, Kalbfleisch S, Li L, Bouet N, Zhou J, Conley R, Chu YS (2016). Multimodality hard-x-ray imaging of a chromosome with nanoscale spatial resolution. Nature Scientific Reports.

[CR91] Yusuf M, Bauer DLV, Lipinski DM, MacLaren RE, Wade-Martins R, Mir KU, Volpi EV (2011). Combining M-FISH and Quantum Dot technology for fast chromosomal assignment of transgenic insertions. BMC Biotechnology.

[CR92] Yusuf M, Parmar N, Bhella GK, Robinson IK (2014). A simple filtration technique for obtaining purified human chromosomes in suspension. BioTechniques.

[CR93] Yusuf M, Chen B, Hashimoto T, Estandarte AK, Thompson GE, Robinson IK (2014). Staining and embedding of human chromosomes for 3D Serial Block Face Scanning Electron Microscopy. BioTechniques.

[CR94] Yusuf M, Millas ALG, Estandarte AKC, Bhella GK, McKean R, Bittencourt E, Robinson IK (2014). Platinum blue staining of cells grown in electrospun scaffolds. BioTechniques.

[CR95] Yusuf M, Zhang F, Chen B, Bhartiya A, Cunnea K, Wagner U, Schwenke J, Robinson IK (2017). Procedures for cryogenic X-ray ptychographic imaging of biological samples. IUCrJ.

[CR96] Yusuf M, Kaneyoshi K, Fukui K, Robinson I (2019). Use of 3D imaging for providing insights into high-order structure of mitotic chromosomes. Chromosoma.

