# Correction to: X-ray ptychography imaging of human chromosomes after low-dose irradiation

**DOI:** 10.1007/s10577-021-09668-z

**Published:** 2021-08-06

**Authors:** Archana Bhartiya, Darren Batey, Silvia Cipiccia, Xiaowen Shi, Christoph Rau, Stanley Botchway, Mohammed Yusuf, Ian K. Robinson

**Affiliations:** 1 London Centre for Nanotechnology, University College, London, UK; 2 Department of Chemistry, University College, London, UK; 3 Research Complex at Harwell, Harwell Campus, Didcot, UK; 4 Diamond Light Source, Harwell Campus, Didcot, UK; 5 Department of Physics, New Mexico State University, Las Cruces, NM 88003 USA; 6 Centre for Regenerative Medicine and Stem Cell Research, Aga Khan University, Karachi, Pakistan; 7 Condensed Matter Physics and Materials Science Division, Brookhaven National Lab, Upton, NY 11973 USA

## Correction to: Chromosome Res (2021) 29:107–126 10.1007/s10577-021-09660-7

The article X-ray Ptychography Imaging of Human Chromosomes After Low-dose Irradiation, written by Archana Bhartiya, Darren Batey, Silvia Cipiccia, Xiaowen Shi, Christoph Rau, Stanley Botchway, Mohammed Yusuf and Ian K. Robinson, was originally published Online First without Open Access. After publication in volume 29, issue 1, pages 107–126 the author decided to opt for Open Choice and to make the article an Open Access publication. Therefore, the copyright of the article has been changed to @The Author(s) 2021 and the article is forthwith distributed under the terms of the Creative Commons Attribution 4.0 International License, which permits use, sharing, adaptation, distribution and reproduction in any medium or format, as long as you give appropriate credit to the original author(s) and the source, provide a link to the Creative Commons licence, and indicate if changes were made. The images or other third party material in this article are included in the article’s Creative Commons licence, unless indicated otherwise in a credit line to the material. If material is not included in the article’s Creative Commons licence and your intended use is not permitted by statutory regulation or exceeds the permitted use, you will need to obtain permission directly from the copyright holder. To view a copy of this licence, visit http://creativecommons.org/licenses/by/4.0/.

The online version of the original article can be found at 10.1007/s10577-021-09660-7.

